# Coffee: Its Therapeutics

**Published:** 1884-04

**Authors:** 


### COFFEE: ITS THERAPEUTICS.

So universally is coffee used nowadays that its relations to our ailments cannot fail to be a necessary study. We give as complete a list of its therapeutics as can be found:

DESIRE for coffee: ANG., *Ars.*, AUR., BRY., Calc. ph., *Chin.*, Colch., *Con.*, Lach., Mosch., Nux mos., Ph. ac., SELEN.

DISLIKE to: Bell., BRY., CALC., Carbo veg., CHAM., Chelid., Chin., Cinnab., Cocc. c., COFF., Dulc., Fluor. ac., Lil. tig, *Lyc.*, Merc., Natr. m., NUX V., Osm., Oxal. ac., PHOS., Rheum, Rhus, *Sabad.*, Spig., *Sul. ac.*

AILMENTS from: Acet. ac., Arg. n., Ars., *Bell.*, *Calc.*, Calc. ph., Camph., CANTH., Caps., *Carbo veg.*, CAUST., CHAM., Cocc., Coloc., HEPAR, IGNAT., IPEC., LYC., Mag. c., Mag. s., Mang., MERC., Millef., Nitr. ac., *Nitrum*, NUX V., *Plat.*, Phos., PULS., *Rhus*, *Sep.*, *Sabin.*, SULPH., Sul. ac., Thuja.

— anxiety from coffee: Cham., Ign., Nux v.

— ill-humor: Calc. ph.

— cough aggr.: Caps., Caust., Cham., Cocc., Ign., Nux v.

— diarrhœa, aggr.: Brom., Caust., Cistus, Cycl., Fluor. ac., Ign., Osm., Oxal ac., Phos., Thuja.

AILMENTS from: headache, aggr.: Arg. n., Arn., Arum tr., Cham., Cocc., Ign., Millef., Nitrum, Nux v.
— stomach (and abdomen) symptoms of, aggr.: Canth., Cham., Ign., Natr. m., Nux v.
— smell of coffee, aggr.: Lach. (headache), Sul. ac. (cough).
— vomiting from coffee: Camph., Phyto.
AMELIORATION from coffee: Ambr., Anac., ARS., Bar., Bell., Bry., Canth., Carbo veg., CHAM., COLOC., Euphor., Glon., Ign., Kali c., Mezer., Phos., Ph. ac., Puls.
— black coffee relieves gastric symptoms: *Brom.*, *Coloc.*
— coffee amel. diarrhœa: Brom., Phos.
— — — headache: Cann. ind., Coloc., Glon., Hyos.

